# New Developments and Future Challenges of Non-Destructive Near-Infrared Spectroscopy Sensors in the Cheese Industry

**DOI:** 10.3390/s26020556

**Published:** 2026-01-14

**Authors:** Maria Tarapoulouzi, Wenyang Jia, Anastasios Koidis

**Affiliations:** 1 Department of Chemistry, Faculty of Pure and Applied Science, University of Cyprus, Nicosia 1678, Cyprus; tarapoulouzi.maria@ucy.ac.cy; 2 Institute for Global Food Security, School of Biological Sciences, Queen’s University Belfast, Belfast BT9 5DL, UK

**Keywords:** NIR sensing technologies, non-invasive testing, spectroscopic quality assessment, cheese quality

## Abstract

Near-infrared (NIR) spectroscopy has emerged as a pivotal non-destructive analytical technique within the cheese industry, offering rapid and precise insights into the chemical composition and quality attributes of various cheese types. This review explores the evolution of NIR spectral sensors, highlighting key technological advancements and their integration into cheese production processes as well as final products already in markets. In addition, the review discusses challenges such as calibration complexities, the influence of sample heterogeneity and the need for robust data and interpretation models through spectroscopy coupled with AI methods. The future potential of NIR spectral sensors, including real-time in-line monitoring and the development of portable devices for on-site analysis, is also examined. This review aims to provide a critical assessment of current NIR spectral sensors and their impact on the cheese industry, offering insights for researchers and industry professionals aiming to enhance quality control and innovation in cheese production, as well as authenticity and fraud studies. The review concludes that the integration of advanced NIR spectroscopy with AI represents a transformative approach for the cheese industry, enabling more accurate, efficient and sustainable quality assessment practices that can strengthen both production consistency and consumer trust.

## 1. Introduction

The integration of non-destructive analytical technologies into food production systems has revolutionised quality control, process optimisation and sustainability in the agri-food sector. Among these technologies, near-infrared (NIR) spectroscopy has emerged as a cornerstone for rapid, reagent-free and environmentally friendly compositional analysis.

NIR spectroscopy originated in the 1960s, initially serving agricultural applications such as grain and forage analysis [1]. Early systems were bulky and limited in sensitivity, requiring extensive sample preparation and skilled operation. Technological advances in the 1980s, including diode-array detectors and Fourier-transform NIR (FT-NIR), improved spectral resolution and enabled real-time measurements, facilitating the transition of NIR spectroscopy from laboratory to industrial use.

Recently, NIR hardware has witnessed exponential advancements, driven by the miniaturisation of optical components and the development of high-sensitivity detectors such as indium gallium arsenide (InGaAs) photodiodes [2]. Portable and handheld NIR devices democratised access to spectral analysis, enabling in-line and on-site measurements [3]. Concurrently, improvements in data processing capabilities, from traditional chemometrics to classical machine learning (ML) and Artificial Intelligence (AI) approaches with cloud computing, transformed raw spectral data into actionable insights. Modern NIR sensors now integrate multivariate calibration models, such as partial least squares regression (PLSR) and support vector machines (SVM), to predict cheese composition with precision, often matching traditional wet chemistry methods such as the Kjeldahl method to determine total protein [4].

The application of NIR spectroscopy in the cheese industry, a sector characterised by stringent quality standards and complex biochemical matrices, has gained significant momentum over the past three decades. Its adoption in cheese production began in the late 1990s, driven by the dairy industry’s need for rapid, non-invasive quality assessment. Early research focused on quantifying major constituents such as moisture, fat and protein in solid and semi-solid cheese matrices [5]. Studies demonstrated the feasibility of NIR for predicting moisture content in low-fat ricotta cheeses, achieving correlation coefficients (R^2^) exceeding 0.95 [4]. These findings catalysed industrial interest, particularly in Europe and North America, where large-scale dairy processors sought to replace time-consuming Kjeldahl and Soxhlet methods for protein and fat determination, respectively, with inline NIR systems.

Nowadays, NIR sensors are embedded at multiple stages of cheese production, from raw milk analysis to final product grading. In curd processing, real-time NIR monitoring of moisture and fat content enables dynamic adjustments to cutting and cooking parameters, reducing batch inconsistencies [6]. During ripening, hyperspectral NIR imaging tracks proteolysis and lipid oxidation, providing insights into flavour development and shelf-life prediction [7,8]. In addition, hyperspectral NIR can detect animal species in cheese [9]. Furthermore, NIR-based sorting systems classify cheese varieties (e.g., Cheddar vs. Gouda) by spectral fingerprints [10] or classify commercial Cheddar cheeses from different brands [11], minimise cross-contamination risks in multi-product facilities and help in surveillance, respectively.

The economic and environmental benefits of NIR adoption are equally compelling. By minimising reagent use and analytical waste, NIR aligns with the dairy sector’s sustainability goals [12]. Process efficiencies, such as reduced energy consumption during drying and optimised yield through precise moisture control, further enhance profitability. Notably, the technology’s non-destructive nature preserves product integrity, a critical advantage for premium artisanal cheeses subject to visual and tactile, texture-based grading [13].

Despite its transformative impact, critical gaps persist in the deployment of NIR sensors for cheese analysis. First, standardisation of spectral protocols remains elusive. Variations in cheese composition (e.g., moisture gradients in aged varieties) and surface irregularities (e.g., rind formation) introduce spectral noise, complicating calibration model generalisability across products and production sites [14]. Second, while NIR excels at quantifying macronutrients, its ability to detect trace contaminants (e.g., mycotoxins) or authenticate geographical origin remains very limited compared to chromatographic or isotopic methods [15,16]. Third, the integration of NIR into fully automated production lines is hindered by latency in real-time data processing, particularly for hyperspectral systems generating terabytes of data per hour [17], as well as speed of analysis factoring conveyor belt speeds [18].

The past five years, however, have seen remarkable strides in addressing these challenges. Advances in hyperspectral imaging now enable spatially resolved spectral analysis, mapping compositional heterogeneity within individual food products [19]. Portable NIR sensors paired with AI-driven edge computing (deep learning models) have reduced latency to milliseconds, facilitating real-time decision-making [20]. Additionally, multi-sensor fusion, combining NIR with Raman, has improved detection limits for contaminants like aflatoxin M1 in milk [21]. Despite these innovations, scalability for small-to-medium enterprises (SMEs) remains a bottleneck, as high costs and technical expertise limit adoption outside large corporations.

Other reviews, such as the review by Silva et al. [14] offers a comprehensive overview of infrared spectroscopic techniques applied to cheese authentication, with emphasis on laboratory-based MIR, NIR and Raman approaches, chemometric methods and authentication challenges. Similarly, Yakubu et al. [22] focus on electronic nose technologies for dairy applications, highlighting sensor design, aroma analysis and pattern-recognition strategies across dairy products. In contrast, the present review provides a distinct and complementary perspective by focusing specifically on the recent emergence and industrial relevance of portable, handheld and miniaturised NIR spectroscopic systems for the cheese sector. Rather than broadly surveying spectroscopic or sensor technologies, this review concentrates on technology readiness, on-site applicability and process integration within the cheese production chain. Therefore, this review aims to critically evaluate the current state of NIR spectral sensors in cheese production, focusing on their technological advancements, operational challenges and future potential. Specifically, it will (a) assess the maturity of NIR systems for diverse cheese matrices and particularly the portable, handheld technological advances, (b) examine persistent barriers such as calibration transferability and cost scalability for SMEs and explore emerging opportunities like AI-driven spectral analysis and multi-sensor fusion. By synthesising recent research (2019–2025), this article provides a roadmap for optimising NIR deployment, scalability and perhaps most importantly, data interpretation, addressing gaps in standardisation and aligning the technology with Industry 4.0 demands in the dairy sector.

In terms of the methodology of the review, the literature search was conducted using major scientific databases, including Scopus and Web of Science, to identify peer-reviewed studies published between 2019 and 2025, relevant to NIR spectroscopy applications in the cheese industry. Searches were performed using targeted keywords related to NIR spectroscopy, cheese, dairy products, authentication and portable or miniaturised analytical systems. Only articles written in English and focused on dairy-related applications of NIR spectroscopy, with particular emphasis on cheese and milk systems, were included. Studies unrelated to dairy matrices, non-peer-reviewed publications, conference abstracts and purely theoretical works without experimental validation were excluded.

## 2. Current Applications of NIR Spectral Sensors in the Cheese Industry

NIR spectral sensors are widely used in the cheese industry for analysing the chemical composition of different cheese types. These sensors provide rapid, non-destructive measurements of essential components such as moisture, fat, protein, lactose, etc. [23,24,25]. Table 1 summarises representative studies in this field. By capturing the unique absorption patterns of these constituents in the near-infrared region, NIR sensors enable real-time monitoring of cheese composition. This helps in ensuring that the cheese meets the desired nutritional and quality standards. For instance, a producer can adjust production processes to ensure consistency in fat content or moisture levels, which directly affect the texture and taste of the cheese [26].

Although several studies listed in Table 1 focus on milk rather than cheese matrices, their relevance to the cheese industry is substantial, as milk composition, authenticity and physicochemical properties directly influence cheese yield, texture, ripening behaviour and final quality. Beyond compositional analysis, NIR spectral sensors are widely applied to assess critical quality attributes such as texture, firmness and flavour-related precursors by detecting subtle structural and chemical variations within the product. These capabilities allow producers to optimise processing conditions and align product characteristics with consumer preferences [28].

NIR spectral sensors also play a key role in quality control by enabling rapid detection of deviations from regulatory and quality specifications, including safety-related parameters such as adulterants and macronutrient levels. Their integration into automated and continuous monitoring systems supports consistent product quality and regulatory compliance throughout production [17,33]. Furthermore, NIR-based monitoring of cheese ripening allows tracking of chemical changes over time, facilitating accurate determination of optimal maturation points, improving batch consistency and reducing waste associated with improper ripening [34,35].

Another sensitive and emerging field of the cheese industry is the cheese products with Protected Designation of Origin (PDO) and Protected Geographical Indication (PGI) schemes, as they play a fundamental role in preserving the heritage, identity and economic value of traditional cheeses produced in specific regions. Examples include Parmigiano Reggiano (Italy), Roquefort (France), Feta (Greece) and Halloumi (Cyprus). These labels are not only markers of quality but also legal instruments that protect producers against imitation and food fraud. However, ensuring compliance with PDO/PGI specifications, such as specific ingredient profiles, production methods and geographical origin, presents ongoing analytical and regulatory challenges, especially in the face of growing international trade and counterfeit risks [36,37].

As NIR sensors allow rapid, in situ measurements of cheese composition without altering or damaging the product, this capability is particularly valuable for PDO/PGI cheeses, where maintaining the integrity of samples is critical due to their high commercial and cultural value [37]. When integrated with advanced chemometrics techniques or AI algorithms, their spectral datasets can be used to develop predictive and classification models capable of identifying authenticity, detecting adulteration and verifying compliance with PDO/PGI specifications. For instance, spectral fingerprints can help differentiate between traditional Halloumi made from a specified blend of sheep and goat milk and imitations made predominantly from cow’s milk [9], an issue of significant regulatory concern since Halloumi’s recent registration as a PDO product within the EU [37,38].

With the global supply chain expanding and instances of food fraud on the rise, ensuring the authenticity of cheese products is more important than ever. NIR spectral sensors provide a powerful tool for detecting adulteration and confirming the authenticity of cheese versus other spectroscopic or chromatographic techniques. By comparing the spectral signatures of a sample with known standards, these sensors can identify any inconsistencies that may indicate fraud or mislabelling. This is particularly useful for premium cheeses, where small deviations in quality or origin can significantly impact market value. NIR sensors can quickly detect adulteration, such as the presence of cheaper oils or non-dairy components, as well as organic vs. non-organic and pasture milk content, helping to protect both consumers and producers from fraud [24,29,32,33,39]. As the cheese industry increasingly seeks sustainable, transparent and digital quality assurance solutions, the application of non-destructive NIR spectral sensors within the PDO/PGI framework offers a promising pathway toward innovation, integrity and consumer trust.

## 3. Technological Advancements in NIR Spectral Sensors

As Table 2 shows, this section discusses the technological advancements in NIR spectral sensors, and a summary of this part is presented there. Primarily, one of the most significant technological advancements in NIR spectral sensors is the improvement in sensor design. This includes innovations such as miniaturisation, which has led to the development of compact, portable NIR devices that can be easily integrated into various stages of the production process.

These smaller, lightweight sensors retain the same analytical capabilities as their larger predecessors, offering more flexibility in how and where they are used. Table 2 provides a representative overview of key studies, highlighting recent technological advancements and benefits from the application and implementation.

Miniaturised NIR spectrometers are compact, lightweight and cost-effective instruments specifically developed to enable efficient on-site or on-farm product analysis. Moreover, increased sensitivity in modern NIR sensors has enhanced their ability to detect even minute changes in chemical composition or quality attributes. This is especially useful in applications such as food production, where precise measurements of moisture, fat, protein and other components are crucial. These improvements in sensor design are making NIR technology more accessible to small and medium-sized enterprises, who can now benefit from high-performance analysis without the need for large, expensive equipment [27,40,41].

As NIR spectral sensors generate large amounts of data, the challenge of processing and interpreting this information effectively has been a focus for recent advancements. The integration of machine learning (ML) into NIR spectroscopy is revolutionising the way data is handled. ML models are being used to improve the accuracy and robustness of calibration models, which are critical for translating raw spectral data into meaningful chemical or physical parameters. ML algorithms, for instance, can analyse vast datasets from NIR sensors, detecting patterns and correlations that may not be immediately evident to human analysts. These models are particularly useful for building predictive tools that can provide real-time feedback during production, improving decision-making and process optimisation. Furthermore, the non-linear and near-black box nature of ML-related methods has gained attention in data robustness, large-scale data management and noise standardisation [44]. The combination of ML with NIR sensors enhances not only the speed of data interpretation but also its accuracy, leading to more reliable and actionable insights [42].

Traditionally, NIR spectroscopy in industries like cheese production involved off-line analysis, where samples were taken to a lab for testing. This method, while accurate, is time-consuming and may not provide real-time insights into the production process. Recent advancements in NIR technology have given rise to in-line and real-time monitoring systems, which are transforming the way quality control is performed. In off-line analysis, samples are removed from the production line and tested separately. This approach requires labour-intensive sampling and delays in obtaining results, which may lead to inefficiencies or delays in adjusting the production process. The latest NIR sensors allow for in-line analysis, where the sensor is directly integrated into the production line. This enables continuous, real-time monitoring of the product’s chemical composition and quality attributes without interrupting production. Real-time data allows producers to make immediate adjustments to ensure product consistency and quality, reducing waste and optimising operational efficiency [6,43].

It must be noted that in-line NIR sensors provide several advantages over off-line methods, including faster response times, fewer human errors and the ability to monitor large volumes of product without the need for destructive sampling (Bhatt et al., 2023) [43]. This is especially beneficial in industries like cheese production, where variables such as moisture and fat content can fluctuate during processing and directly impact the quality of the final product. It must be said, however, that the right sampling technique is very important for the prediction outcome [45].

## 4. Challenges and Limitations in Using NIR Spectral Sensors in the Cheese Industry for In-Line Analysis and Real-Time Monitoring

NIR spectral sensors in the cheese industry have been implemented for in-line analysis and real-time quality assessment monitoring by offering rapid, non-destructive scanning of composition (e.g., moisture, fat, protein) and the fingerprint of cheese products. However, despite these advantages, several challenges are supposed to be considered before comprehensively applying NIR in production environments. Key challenges include correct sampling techniques and spectral acquisition, the transferability of robust calibration models across instruments and production sites, the high variability inherent to cheese, which leads to difficulties in interpreting the spectral data, and practical issues of cost and accessibility when focusing on small-sized producers. Representative studies in Table 3 show a summary of these challenges and limitations and each point is then critically analysed in the next sessions.

### 4.1. Calibration Complexity and Model Transferability

As has been proven for decades, the Partial Least Squares (PLS) method is used for NIR calibration because of its strong explanatory ability based on the linear methodology, but it acquires low accuracy in some cases [17]. Also, studies show that models built on limited sample sets often fail to generalise, whereas hundreds of samples covering the full variability of cheese composition are needed for reliable prediction [22]. Even then, a model calibrated for one cheese variety or process often fails on others without recalibration. Advanced machine learning models have been explored to improve accuracy, such as a series of neural networks (NNs) that can capture complex spectral patterns and have outperformed conventional chemometrics methods in specific cases [53]. These approaches (including deep learning) show improvement in modelling the intricate relationships in NIR data but also demand hyperparameter optimisation and careful validation to avoid overfitting [47,54].

Apart from the algorithm, an emerging challenge is that calibration models tend to be instrument- and condition-specific. A model developed on one spectrometer or production line often underperforms on another due to hardware and sample preparation differences. Several strategies were proposed to address this by generating a robust calibration model or adjusting the model and spectra: making robust calibrations, adjusting calibrations and adjusting spectra [55]. Ref. [46] proposed a calibration transfer method based on result correction and transfer learning; Yang et al. [49] provided a deep learning approach for calibration transfer analysis and evaluated different calibration transfer strategies to generate a calibration transfer model [48]. For future development, explainable AI is one of the methods to balance the model complexity and robustness when transferring the calibration model by pinpointing specific spectral regions and illuminating the reasoning behind model outputs.

Another practical measure to address calibration drift and facilitate model transferability is recalibrating the model using stable reference cheese materials. In this approach, freeze-dried cheese powders serve as physical calibration standards, providing a consistent spectral benchmark for adjusting NIR models. These materials preserve the cheese matrix in a homogeneous, moisture-free form, which improves spectral consistency [52]. By routinely measuring reference samples, systematic shifts in instrument response or model bias can be detected and corrected. This approach could also be applied in conjunction with unsupervised techniques, as the reference spectra offer anchor points to realign models without additional wet-chemistry analyses. Incorporating freeze-dried standards into the calibration protocol thus helps maintain model accuracy over time and supports the reliable transfer of NIR models across diverse instruments or processing conditions.

### 4.2. Sample Variability and Data Interpretation

Besides implementing a calibration model from the perspective of algorithm and hardware adaptability, the heterogeneous nature of cheese introduces substantial variability in NIR measurements. Differences in composition and texture can shift the spectrum across different cheeses and batches [51]. Cheese variety is a significant source of spectral variation, factors such as seasonal milk changes, process conditions and ripening stage all influence the spectra, often necessitating product-specific models [31,52]. A near-standardised sampling protocol could help enhance the stability of sample variability beyond the composition and temperature. Moreover, in the actual scanning environment, physical scattering effects and temperature fluctuations further mislead measurements. Some chemometric methods (e.g., scatter corrections, derivatives) could help reduce these types of interferences, but the uncertainty from the cheese matrix is inevitable.

Also, interpreting the spectral data in the real world presents another challenge. Unlike mid-infrared, near-infrared data are broad and overlapping, so individual chemical features cannot be directly identified. Multivariate models are needed to precisely estimate the contributions of water, fat and protein, but their outputs appear as a “black box”. Feature selection methods are important for improving the interpretability in NIR cheese analysis by identifying and using the most informative wavelengths. This approach yields simpler and more robust models across different samples, since irrelevant variations are minimised. Moreover, limiting the predictor set to relevant features can facilitate periodic model recalibration; with fewer variables to adjust, updating the calibration with new reference data becomes more straightforward [54].

### 4.3. Cost and Accessibility for Small Producers

Economic considerations remain a major barrier to the adoption of in-line NIR spectroscopy, particularly for small-scale cheese producers. While high-performance in-line systems are readily adopted by large dairy companies as efficiency-enhancing tools, their acquisition and operational requirements often exceed the financial capacity of artisanal producers. Additional costs related to maintenance, calibration and specialised technical expertise further increase the overall burden.

Recent advances in miniaturised and handheld NIR devices offer a more accessible alternative, as these systems are available at a fraction of the cost of traditional benchtop instruments. Handheld spectrometers have demonstrated the ability to predict protein content and other quality attributes in cheese, enabling rapid on-site analysis with simplified operation and reduced investment requirements [26,35]. However, such devices typically operate over narrower wavelength ranges and with lower spectral resolution, resulting in less comprehensive information compared with industrial or laboratory-based systems [40,56,57].

In terms of the cost of NIR systems, it strongly depends on instrument configuration and performance, with handheld and basic portable units representing a lower entry threshold for SMEs, whereas advanced portable and high-end FT-NIR systems entail substantially higher total ownership costs, including software, calibration, training and maintenance [58,59]. Despite these challenges, ongoing technological developments are steadily reducing costs and improving usability. Continued progress in adaptive calibration strategies, data interpretation and affordable high-resolution sensors is expected to further bridge the gap between laboratory capability and industrial deployment, supporting broader adoption of NIR spectroscopy across cheese production scales.

## 5. Future Potential and Innovations in NIR Technology

As NIR technology continues to evolve, its applications in various industries, including cheese production, are expanding. This emerging landscape is driven by advancements in real-time monitoring, portable devices, AI integration and the exploration of new areas where NIR technology could have significant impacts. The integration of NIR sensors directly into production lines for continuous, real-time monitoring is one of the most promising advancements in NIR technology. Real-time in-line monitoring allows for constant analysis of key quality parameters, such as moisture, fat content and protein levels, without interrupting the production process. This continuous flow of data enables producers to make instant adjustments to maintain product consistency and optimise production efficiency. In cheese production, for example, the ability to monitor critical factors like moisture and fat in real time can prevent costly deviations from product specifications and reduce waste [60]. Real-time data can also help maintain ideal conditions for cheese ripening and maturation, ensuring that the final product consistently meets desired standards. As this technology advances, NIR sensors could become a standard feature in fully automated production environments, where quality control (process analytical technology, PAT) is seamlessly integrated into the process [6,54].

Another key innovation in NIR technology is the development of portable and handheld NIR devices, which are becoming increasingly popular across various industries [40,55,61,62]. These devices offer the flexibility of on-site analysis at multiple stages of the production and supply chain, eliminating the need for laboratory-based testing. In the cheese industry, handheld NIR sensors can be used to assess the quality of raw materials before they enter the production process, allowing producers to make informed decisions about ingredient selection. Additionally, portable devices can be used for spot checks at different stages of production, ensuring that products meet quality standards without requiring extensive sampling or delays. The growing accessibility of these compact, portable NIR devices also opens opportunities for quality control in other parts of the supply chain, such as during transportation and storage. By using handheld NIR sensors to monitor cheese during transit, producers can ensure that optimal conditions are maintained, minimising the risk of spoilage or quality degradation [26].

The combination of NIR technology with AI and ML holds vast potential for revolutionising data analysis, predictive maintenance and process optimisation. AI-driven models can process the vast amounts of data generated by NIR sensors more effectively, uncovering patterns and insights that might otherwise be missed [44]. In cheese production, AI and ML can be integrated into NIR systems to create predictive models for product quality. For example, these models can analyse data from NIR sensors in real time to forecast the optimal conditions for cheese ripening or predict when certain quality parameters are likely to drift out of specification. This enables producers to take proactive measures, optimising the production process and preventing quality issues before they occur. Moreover, AI models can be used for predictive maintenance of the equipment itself. By continuously monitoring the performance of NIR sensors and other production machinery, AI algorithms can identify signs of wear and tear or potential breakdowns before they happen, reducing downtime and maintenance costs [42,63].

Regarding new areas of application, the potential applications of NIR technology in the cheese industry continue to grow and several emerging areas hold promise for future development. In terms of advanced flavour profiling, NIR sensors could be used to analyse the chemical components that contribute to cheese flavour, providing deeper insights into how specific conditions during production and maturation influence taste. This could lead to the development of more precise methods for controlling flavour characteristics in different cheese varieties. Moreover, NIR technology may also play a role in sustainability efforts within the cheese industry by monitoring factors like water usage or energy efficiency during production. Sensors could help identify areas where processes can be optimised to reduce resource consumption. Furthermore, NIR sensors might be integrated into packaging systems to monitor the quality of cheese throughout its shelf life. This could include detecting signs of spoilage or changes in moisture levels, allowing retailers and consumers to assess product freshness in real time [22,64].

## 6. Conclusions

NIR spectroscopy has established itself as a robust analytical platform for characterising cheese matrices, offering rapid, non-destructive quantification of compositional and structural attributes relevant to PDO/PGI authentication. The collective evidence reviewed here indicates that the integration of high-resolution spectral instrumentation with chemometric and machine-learning pipelines is reshaping NIR from a supportive laboratory technique into a viable tool for in-process and supply-chain-level verification. This shift reflects a deeper understanding of how spectral signatures encode microstructural, biochemical and geographical markers intrinsic to protected cheeses.

At the same time, the current body of work exposes several methodological constraints that limit large-scale deployment. Calibration transfer across instruments and production environments remains insufficiently resolved, and model robustness is still challenged by heterogeneity in raw materials, seasonal variability and manufacturing practices. The absence of standardised, interoperable spectral databases for PDO/PGI cheeses further restricts the development of generalizable classification and regression models.

Future progress will depend on advancing domain-adaptation strategies, multimodal sensor fusion and machine-learning architectures capable of handling high-dimensional, non-linear spectral variation. Equally important will be the development of portable, real-time NIR systems with embedded analytics that can operate reliably under industrial conditions. Coordinated efforts among research laboratories, regulatory authorities and producers will be essential to establish harmonised protocols, shared spectral repositories and validation frameworks. These steps will determine the extent to which NIR spectroscopy can evolve into a fully operational technology for safeguarding authenticity, enhancing process control and strengthening data-driven quality management across PDO/PGI cheese supply chains.

## Figures and Tables

**Table 1:** Selected/representative studies (2017–2024) performed with recent systems (portable/handheld/miniature) NIR spectroscopy.

Application/ Objective	Type of Innovative Spectroscopic Setup	Wavelength Range (nm)	Significant Subregion(s) (nm)	Reference
Detection/quantification of melamine adulteration in milk powder	microPHAZIR (GP 4.0), microNIR2200	microPHAZIR: 1600–2400, microNIR2200: 1128–2162	-	[27]
Predicting elastic modulus of the gel (G’) of curd during milk coagulation, monitoring cheese curd hardness	Multifibre optical probe (near-infrared light scattering, inline fibre-optic sensor)	870, 880 and 890	880	[28]
Authentication/classification of organic milk vs. non-organic and pasture-milk	ultra-compact spectrometer, Micro-NIR 1700	908–1676	1220–1390	[29]
Detection of low concentrations of nitrogen-based adulterants in whey protein powder	Handheld NIR-S-G1	900–1700	950–1650	[30]
On-farm analysis of raw milk composition (fat, protein, lactose)	Miniaturised MEMS NIR sensors (Fabry-Pérot Interferometer-based)	Transmission: 1100–1400, 1700–2000, 2200–2500, Reflection: 1700–2000	1700–2000 in transmission mode	[24]
In situ quality control (fat, protein and solids-non-fat) in fresh raw milk	Handheld portable NIR spectrometer (MicroPhazir™)	1600–2400	-	[23]
Milk macronutrient determination in commercial samples	Two pocket-size NIR spectrometers: SCiO and NeoSpectra	SCiO: 740–1070, NeoSpectra: 1350–2558	full range	[25]
Discrimination of Halloumi cheese by season of milk collection (i.e., detecting seasonal variations)	Portable miniature NIR, MicroNIR™ 1700	910–1676	1100–1639	[31]
On-site authentication of buffalo milk via NIR screening	Portable MicroNIR OnSite-W, Viavi	950–1650	full range	[32]

**Table 2:** The technological advancements in NIR spectral sensors in the last 5 years, as shown in representative studies.

Types of Advancement	Description	Aims	Benefits/Applications	References
Hardware-driven advancements				
Miniaturisation of sensors	Development of compact, portable NIR devices	Retain analytical capabilities of larger instruments	On-site and on-farm analysis, flexible integration into production lines, cost-effective for SMEs	[27,40,41]
Increased sensitivity	Modern sensors can detect small changes	Precious chemical composition or quality attributes	Precise measurement of moisture, fat, protein and other components; enhanced food quality control	[27,40]
Accessible and cost-effective instruments	Smaller, portable sensors	High-performance NIR available to SMEs	Broader adoption in food industries, including cheese production	[40,41]
Data-driven advancements				
Integration of AI and ML	AI/ML models improve calibration	Analyse large spectral datasets, detect hidden patterns	Real-time predictive tools, improved accuracy and robustness, process optimisation	[42]
In-line/real-time monitoring	Sensors integrated directly into production lines rather than off-line lab testing		Continuous monitoring, faster response, reduced human error	[7,43]

**Table 3:** Challenges and Limitations in Using NIR Spectral Sensors for In-line Cheese Analysis and Real-time Monitoring.

Challenge/Limitation	Description/Cause	Impact on NIR Performance	Proposed/Emerging Solutions	References
Calibration complexity and model transferability	Calibration models (e.g., PLS) require large, diverse datasets; models are often instrument- and condition-specific.	Reduced prediction accuracy when models are applied to different cheese types or instruments.	Advanced ML for better pattern capture; calibration transfer methods (transfer learning, result correction); explainable AI for interpretability; use of freeze-dried reference cheese powders for recalibration.	[17,22,46,47,48,49,50]
Sample variability and spectral interpretation	Cheese composition varies by type, milk season, ripening and processing; physical scattering and temperature shifts distort spectra.	Spectral variability reduces model robustness and interpretability; overlapping NIR bands obscure specific chemical signals.	Scatter corrections and derivatives; feature selection methods to isolate informative wavelengths; development of product-specific models; use of reduced predictor sets to simplify recalibration.	[31,51,52]
Cost and accessibility for small producers	In-line NIR spectrometers are costly and require expert maintenance; handheld devices have limited range and resolution.	Limits adoption by small/medium cheesemakers; potential trade-off between affordability and analytical power.	Adoption of portable and miniaturised NIR devices; ongoing improvements in usability and affordability; gradual reduction in cost through technological development.	[26,35,40]

## Data Availability

No new data were created or analyzed in this study. Data sharing is not applicable to this article.
